# Associations between physical growth and general cognitive functioning in international adoptees from Eastern Europe at 30 months post-arrival

**DOI:** 10.1186/s11689-015-9132-7

**Published:** 2015-11-15

**Authors:** Maria G. Kroupina, Judith K. Eckerle, Anita J. Fuglestad, Liza Toemen, Stephanie Moberg, John H. Himes, Bradley S. Miller, Anna Petryk, Dana E. Johnson

**Affiliations:** Department of Pediatrics, University of Minnesota Masonic Children’s Hospital, Minneapolis, MN 55454 USA; Center for Neurobehavioral Development, University of Minnesota Masonic Children’s Hospital, Minneapolis, MN 55454 USA; Maastricht University, Maastricht, The Netherlands; Division of Epidemiology and Community Health, University of Minnesota, Minneapolis, MN 55454 USA; Department of Psychology, University of North Florida, Jacksonville, FL 32224 USA; Present address: University of Minnesota, 717 Delaware Street SE, Room 361, Minneapolis, MN 55414 USA

**Keywords:** Cognitive functioning, Physical growth, Growth hormone system, International adoption, Early adversity

## Abstract

**Background:**

Internationally adopted children have often experienced early adversity and growth suppression as a consequence of institutional care. Furthermore, these children are at risk for impaired cognitive development due to their early adverse experiences. This study examined the association between physical growth, the growth hormone (GH) system, and general cognitive functioning post-adoption. Based on previous research, we expected to find that a child’s initial physical growth status and normalization of the growth hormone-insulin-like growth factor 1 (GH-IGF-1) axis would be positive predictors of general cognitive functioning.

**Methods:**

Post-institutionalized children (*n* = 46) adopted from Eastern Europe were seen approximately 1 month after their arrival into the USA to determine baseline measurements. They were seen again 6 and 30 months later for two follow-up sessions. Measures included anthropometry, insulin-like growth factor-1 (IGF-1), IGF binding protein-3 (IGFBP-3), Mullen Scales of Early Learning, and Stanford-Binet Intelligence Scales. Information about parental education was also collected.

**Results:**

We found that a child’s general cognitive functioning at 30 months post-adoption was predicted by their general developmental scores at 6 months post-adoption, their initial height status, and markers of the growth hormone system. Children with lower initial IGFBP-3 standard deviation (SD) scores had higher verbal IQ scores at 30 months. Furthermore, a child’s initial height was found to be a significant positive predictor of non-verbal IQ.

**Conclusions:**

These results suggest an association between a child’s suppressed physical growth in response to early adversity and alterations in GH system functioning and subsequent recovery in cognitive functioning.

## Background

There are 163 million children in 93 countries without permanent parents [1, 2]. Of these, 2–8 million reside in institutional care where they often face social and emotional deprivation, poor nutrition, and inadequate medical care. Although internationally adopted children comprise a relatively small group within this population, only 8668 adoptions into the USA in 2012 [3], they provide us with a unique research opportunity. For many of these children, adoption is a major intervention that attenuates the far-reaching impact of early adversity and leads to enduring, positive gains in multiple domains, including physical growth and general cognitive functioning. Therefore, the period of adversity experienced by internationally adopted children is demarcated, ending at the time of their removal from institutional care. Consequently, this allows us to analyze multiple factors contributing to positive post-adoption changes in physical status and in general cognitive functioning. Our goal in analyzing these factors is to enable clinicians and researchers to explore how adversity experienced early in life translates into long-term neurodevelopmental outcomes.

### Impact of early adversity on physical growth

Psychosocial deprivation negatively impacts all three major physical growth parameters: height, weight, and head circumference [4, 5]. The etiology of growth failure can be largely attributed to malnutrition, infectious diseases, and maltreatment/neglect [6]. Caloric failure, leading to malnutrition, has been identified as a significant component of growth failure in early infancy [5]. Chronic infectious diseases and chronic stress divert substrate away from growth and towards the inflammatory response throughout childhood, and particularly, during periods of rapid physical growth [7]. Growth suppression resulting from maltreatment/neglect leads to changes in the growth hormone-insulin-like growth factor-I axis (GH-IGF-1 axis), which plays a bigger role after 18 to 24 months of age [8]. IGF-1, a major mediator of the growth-promoting effects of growth hormone, and IGFBP-3, a central binding protein for IGF-1 that carries IGF-1 in the bloodstream, have been used to assess growth hormone excess or deficiency [5]. Emergent research using animal models [9] and extrapolations of studies investigating early adversity in humans suggest that IGF-1 is a key mediator of the impact of early experience on brain development [7, 10].

Physical growth in institutionalized children has been shown to improve following placement in an environment that provides better nutrition and improved quality of caregiving. This can be observed both when children are placed in foster care [5] and when children are adopted [11]. Catch-up in height and weight in post-institutionalized (PI) children usually nears completion, while catch-up in occipital frontal circumference (OFC) size tends to be slower and remains incomplete [12]. However, catch-up rates can vary. For example, children who are older at the time of adoption often exhibit a greater delay in physical growth and less complete catch-up overall [11]. Predictors of greater catch-up growth post-adoption include: lower baseline auxological parameters, placement in an adoptive family before 12 months of age, and higher quality of caregiving [5].

Changes in the growth hormone system are also observed following placement in a more nurturing environment. A previous study done by our research team indicated that PI children had lower levels of IGF-1 and IGFBP-3 upon their arrival into the USA in comparison to reference values, suggesting a suppression of the GH-IGF-1 axis [7]. Recovery of the GH-IGF-1 axis, in terms of improving growth factors, was observed in the majority of children during the 6-month interval after adoption [10].

### Impact of early adversity on general cognitive functioning

Psychosocial deprivation caused by institutional care negatively affects general cognitive functioning. In some cases, these deficits are as severe as those seen in children with borderline mental retardation [13–15]. These deficits are the result of factors associated with institutional care including: unfavorable caregiver-to-child ratios; highly regimented routines; impoverished sensory, cognitive, and linguistic stimulation; and unresponsive caregiving practices [13]. However, research suggests that recovery from these deficits, as with those in physical growth, is possible when placed in a more nurturing environment with adequate sensory and social stimulation [13]. Imaging studies with this population found that cortical white matter was no different for children placed in foster care than never-institutionalized children, but was significantly smaller for children not randomized to receive foster care [16]. Given these findings, it was suggested that a high-quality family environment could support more normative trajectories of white matter growth and improve connectivity during this period of growth [16]. Institutionalized children who are placed in optimal environments also show significant and enduring gains in general cognitive function [13, 17].

Importantly, the gains in general cognitive functioning brought about by environmental changes vary. It seems that the duration and degree of initial deprivation are implicated in the varying levels of impairment and subsequent catch-up in a child’s cognitive functioning [6, 18]. For example, children who were younger when they were placed in foster care showed enhanced cognitive gains. Conversely, children with disabilities required greater exposure to sensitive caregiving practices than healthy children in order to show catch-up in cognitive functioning [19]. The most complete catch-up in the cognitive domain for institutionalized children is seen in those who are adopted out of their native country and placed in a family environment in western countries with a higher standard of living (e.g., US, UK, and Western Europe) [12, 18]. However, the determinants of these positive changes are not fully understood.

### Relationship between physical growth and general cognitive functioning

Impaired growth in institutionalized children has previously been associated with poor general cognitive functioning [19]. Recently, a parallel between the changes in growth and the changes in cognitive development post placement in a foster care family has also been suggested [5]. The Bucharest Early Intervention Project (BEIP) provided important data regarding the relationship between changes in physical growth status and general cognitive functioning. In this project, select children under Romanian institutionalized care were placed in foster care at random. Analyses among the group of children remaining in the institutions, the group of children placed in foster care, and a group of children living with their biological families revealed an association between height and cognitive status. Additional predictors of cognitive abilities included baseline developmental status, birth weight, and catch-up growth in height. Catch-up growth in height observed in those children placed in foster care was an independent predictor of cognitive abilities at follow-up at 42 and 54 months of age [13]. It was suggested that changes in the GH system potentially account for this association between catch-up height and the positive changes in general cognitive functioning post adversity.

Recent data indicate that IGF-1 is antiapoptotic, leading to an upregulation of synapses, and is important for synaptic development [20]. IGF-1 is also very important for early brain development, including myelination and survival of cerebellar neurons [20]. The potential interaction between the GH-IGF-1 axis and cognitive development is supported by findings in several different populations. Children with chromosomal syndromes have benefited from treatment with GH, resulting in improvement in both height and cognitive scores [21]. Additionally, IGF-1 levels were positively correlated with intelligence in healthy 8- to 9-year-old children [22]. In patients with isolated growth hormone deficiency, white matter abnormalities in the corpus callosum and corticospinal tract and reduced thalamic and globus pallidum volumes relate to deficits in cognitive function and motor performance [20]. Relatedly, previous imaging studies of post-institutionalized children indicated reduced white and gray matter volume and white matter integrity [16, 23]. Given these findings and the physical growth failure and poor cognitive performance of many PI children, it is important to consider the impact of growth hormone deficiency on brain structure and cognitive function.

### Hypotheses

Previous studies have demonstrated the magnitude and scope of the impact that institutional care has on general cognitive functioning and physical growth [4, 8, 15]. Recent data have suggested associations between physical growth and cognitive outcomes may be driven by changes in the GH system. However, this association has not yet been explicitly investigated. The current study seeks to fill this gap and to investigate the interaction between post-adoption changes in physical growth and the growth hormone system and their impact on general cognitive outcomes during the first years of a child’s placement in a highly enriched environment.

Based on the findings of catch-up growth at 6 months post-adoption [10], we expect that at 30 months, catch-up growth will correspond to the child’s age and the initial severity of growth failure at the time of arrival. Furthermore, we hypothesize that a better initial height status will be a positive predictor of cognitive status at 30 months post-arrival. We expect initial status and normalization of the GH axis to be positive predictors for a child’s cognitive and physical growth statuses. In agreement with previous research, we expect to find length of deprivation, as indicated by age at adoption, to be a negative predictor for cognitive development.

## Methods

### Participants

All participants initially included (*N* = 122) were part of a larger longitudinal study on growth, neuroendocrine changes, and cognitive development following international adoption [7, 10]. Participants were recruited when their initial medical evaluation was scheduled with their parents within 1 month of their arrival in the USA. Parents provided written consent for their child’s data to be used in the study. The Institutional Review Board of the University of Minnesota between March 2004 and March 2007 approved this study.

Participants whose data were used in analysis (*N* = 46) were between 8.8 and 45.6 months of age (*M* = 18.9 months). This subsample of participants was created as a result of those participants who were able to be located and were available and healthy enough to complete neurodevelopmental testing at the 30 months post-adoption appointment. All children were adopted into the USA from Eastern Europe. The participants’ countries of origin included: Russia (74 %), Kazakhstan (15 %), and Ukraine (11 %). Most of the children (72 %) had spent more than 75 % of their lives in institutions or hospitals. Within this group, 94 % of children had not lived with their birth parents at all.

After the initial appointment, participants were invited to both 6- and 30-month follow-up appointments. Children with a high risk for fetal alcohol spectrum disorder (FASD) (*N* = 8) were excluded from cognitive assessment in this study because of the major effects FASD can have on neurocognitive development. Using FASD analysis software [24], pictures of the children were analyzed and the risk of FASD was evaluated (face III and VI). The final subsample (*N* = 46) did not differ statistically from the whole group (not including children with suspected FASD) on baseline age, height, weight, OFC, or growth factor or neurodevelopmental status.

### Procedure

The baseline assessment took place within a month after arrival into the USA. The follow-up assessments took place 6 and 30 months later. At the first and second sessions, anthropometry data were collected, blood was obtained through venipuncture, and the Mullen Scales of Early Learning [25] was administered. At the third session, anthropometry data were collected and the Stanford-Binet Intelligence Scales [26] was administered.

### Measures

#### Physical growth measure

Anthropometry data were collected from medical records at the International Adoption Clinic where trained staff followed standardized measurement protocols. Height or supine length (under 24 months of age) and OFC measurements were obtained by calculating an average of triplicate measurements. Weight was obtained by a single measurement. Age- and gender-specific standard deviation (SD) values for OFC were estimated from previous data [27]. Age- and gender-specific *Z*-scores for length and weight were calculated based on the CDC 2000 growth charts using Epi Info 3.3 [28]

#### Growth factor measure

Blood was collected through venipuncture. Serum (2.5 ml) was obtained and stored at −20 °C until shipping. IGF-1 and IGFBP-3 were quantified by radioimmunoassay performed by Esoterix, Inc. and reported as actual values and SD scores. IGF-1 and IGFBP-3 levels were obtained at baseline and at 6 months. They were previously reported [10].

#### Initial measure of general cognitive development

The Mullen Scales of Early Learning was administered during the first two visits. At the initial baseline appointment, receptive and expressive language assessment tests were not administered. The tests are language-specific and participants had only recently been exposed to English. Raw scores on the remaining subscales were converted to *T*-scores, with a mean of 50 and standard deviation of 10 [25]. For analysis, we averaged the scores on the visual reception and fine motor subscales and created a combined score. At the 6-month follow-up, all four subscales were administered (visual reception, fine motor, receptive language, and expressive language). *T*-scores on the four subscales were obtained and summed to calculate the Mullen Early Learning Composite Score, with a mean of 100 and a standard deviation of 15. Scores below 85 (−1 SD) on the composite score were considered below average.

#### General cognitive development at 30 months

At the 30-month follow-up, the Stanford-Binet Intelligence Scales (fifth edition) was administered to assess intelligence and cognitive abilities [26]. From this assessment, verbal and non-verbal IQ scores were obtained. The full-scale IQ score was then calculated by summing the verbal IQ and non-verbal IQ scores. All scores were converted to normalized, standard scores with means of 100 and standard deviations of 15. Scores below 85 (−1 SD) were considered below average.

#### Parental education

Maternal and paternal levels of education were investigated separately as categorical variables ranging from completion of high school through advanced and professional degrees.

### Statistical analysis

Statistical analysis was performed using SPSS software version 21. Changes in means for anthropometry data and growth factors were compared using paired *t* tests. To identify predictors of neurocognitive outcomes at 30 months post-adoption, a Spearman’s rank correlation coefficient was computed. The variables examined were as follows: age at adoption, physical growth status, growth hormone factors, and cognitive scores at 6 months follow-up. Potential covariates with unadjusted correlations with outcome variables with *p* values of <0.05 were considered for linear regression analyses. Linear regression analysis was also carried out to establish whether changes in anthropometry data and growth factors predicted cognitive scores at 30 months. The categorical parental education variables as a block did not enter the regression models as statistically significant contributors to the developmental outcomes nor did they modify the partial regression coefficients of the other significant covariates sufficiently to suggest appreciable moderation or mediation of main effects.

## Results

### Growth hormone system

Descriptive data for growth, IGF-1, IGFBP-3, and cognitive scores are shown in Table 1, measured at baseline, 6 and 30 months follow-up. At 6 months, there was statistically significant improvement in means for height, weight, and OFC in comparison to baseline values. The positive change in the levels of IGFBP-3 was also statistically significant. Although there was an improvement in the mean for IGF-1 levels at the 6-month assessment, it was not statistically significant. At the 30-month assessment, there was a significant improvement in the means for height and weight (Table 1), but not for OFC, when compared to the respective means at the 6-month assessment.

**Table 1:** Descriptive statistics for the subsample, and means and SD for all variables

Descriptive statistics						
Age at adoption (months)	18.9 (range 8.8–45.6)					
Sex (*n*)	Males: 23		Females: 23			
Country of origin (%)	Russia: 74		Kazakhstan: 15		Ukraine: 11	
	Baseline *M* (SD)	*N*	6-month follow-up	*N*	30-month follow-up	*N*
Anthropometry (*Z*-scores)						
Height	−1.17 (1.00)	46	−0.48 (0.97)***	46	0.00 (1.00)***	46
Weight	−1.60 (1.29)	46	−0.69 (1.10)***	46	−0.08 (1.09)***	46
OFC	−1.02 (1.10)	46	−0.50 (1.29))***	46	−0.46 (1.49)	46
IGF-1 (SD)	−1.09 (1.41)	39	−0.81 (1.51)	39	–	
IGFBP-3 (SD)	−0.86 (1.02)	41	−0.43 (1.01)**	41	–	
Mullen Scales of Early Learning (*T*-scores)						
Visual reception	36.33 (10.85)	42	43.02 (12.51)**	42	–	
Fine motor	39.61(10.57)	44	46.20 (12.22)***	44	–	
Receptive language	–		39.16 (9.57)	45	–	
Expressive language	–		41.07 (9.42)	45	–	
Early learning composite standard score	–		85.87 (15.12)	45	–	
Stanford-Binet Intelligence scales (standard scores)						
Non-verbal IQ	–		–		107.48 (13.38)	46
Verbal IQ	–		–		105.22 (14.92)	46
Full-scale IQ	–		–		106.49 (13.94)	46

Changes in means for anthropometry data and growth factors were compared using paired sample *t* tests, 6-month follow-up was compared with baseline and 30-month follow-up with 6-month follow-up

**p < 0.01; ***p < 0.001

#### Predictors of growth catch-up at the 30-month follow-up

We explored predictors of changes in growth in linear regression models (Table 2). Height at arrival and weight change over 30 months were positive significant predictors for height change over 30 months (*R*^2^ = 0.338, *F*(2,45) = 10.977, *p* < 0.001). The more weight the children gained, the more height change they showed. The children who were smaller on arrival showed more catch-up growth.

**Table 2:** Summary of regression analysis physical growth status

Height change over 30 months (*R* ^2^ = 0.338, *F*(2,45) = 10.977, *p* < 0.001)					
Variable	*B*	SE (B)	*β*	*t*	*p*
Weight change over 30 months	0.295	0.081	0.458	3.635	0.001
Height-for-age at arrival	−0.201	0.089	−0.286	−2.265	0.029
Weight change over 30 months (*R* ^2^ = 0.471, *F*(2,45) = 19.175, *p* < 0.001)					
Variable	*B*	SE (B)	*β*	*t*	*p*
Weight-for-age at arrival	−0.499	0.095	−0.581	−5.236	0.000
Age at arrival	−0.044	0.014	−0.343	−3.091	0.003

Weight change over 30 months was predicted by weight at arrival and age at arrival (*R*^2^ = 0.471, *F*(2,45) = 19.175, *p* < 0.001). Again, the younger children and the children with a lower weight at the time of arrival showed more catch-up growth. Initial growth factors were not independent predictors of growth at 30 months after arrival or catch-up growth, consistent with our previous findings. Gender was not an independent predictor of growth at 30 months or changes in growth*.*

### Predictors of cognitive outcomes at 30-months follow-up

At 30 months post-arrival, 91.3 % of children had verbal and non-verbal IQ scores in the normal range. In order to determine which factors would be included in regression analysis to analyze predictors of cognitive outcomes at 30 months follow-up, Spearman’s rank correlations were computed (Table 3). Verbal IQ at 30 months was correlated with the following: age at arrival, IGF binding protein-3 at arrival, Mullen at 6 months, OFC at arrival, and OFC change over 6 months. Due to limited degrees of freedom, we excluded OFC at arrival from the regression analysis and instead used change in OFC over 6 months. Analysis with the four predictors in a linear regression model (Table 4) showed a significant model, *R*^2^ = 0.365, *F*(4,39)=, *p* = 0.001. IGFBP-3 was a negative predictor, while Mullen at 6 months and OFC change over 6 months were both positive predictors. Age at arrival was not significantly associated with verbal IQ at 30 months.

**Table 3:** Correlations with verbal and non-verbal IQ at 30 months follow-up

Variable	*n*	Verbal IQ		Non-verbal IQ	
		Spearman’s rho	*p*	Spearman’s rho	*p*
Age at arrival	46	−0.37	0.006^a^	−0.36	0.007^a^
Mullen at 6 months	45	0.25	0.050	0.44	0.001^a^
OFC at arrival	46	0.34	0.010^b^	0.25	0.050
OFC change at 6 months	46	0.33	0.014^b^	0.19	0.099
OFC change over 30 months	46	0.21	0.172	0.11	0.454
Weight-for-age at arrival	46	0.13	0.203	0.20	0.093
Weight change over 6 months	46	0.21	0.081	−0.03	0.431
Weight change over 30 months	46	0.15	0.334	−0.03	0.849
Height-for-age at arrival	46	0.19	0.105	0.32	0.016^b^
Height change over 6 months	46	0.20	0.090	0.06	0.338
Height change over 30 months	46	0.06	0.711	−0.16	0.299
IGF-1 SD score at arrival	40	−0.16	0.156	−0.025	0.439
IGF-1 change over 6 months	39	0.16	0.164	0.10	0.561
IGFBP-3 SD score at arrival	42	−0.290	0.031^b^	−0.10	0.273
IGFBP-3 change over 6 months	41	0.17	0.139	0.09	0.294

^a^Correlation is significant at the 0.01 level (one-tailed)

^b^Correlation is significant at the 0.05 level (one-tailed)

**Table 4:** Summary of regression analysis neurodevelopmental status

Variable	Verbal IQ (*R* ^2^ = 0.365, *F*(4,39) = 5.614, *p* = 0.001)				
	*B*	SE (B)	*β*	*t*	*p*
Age at arrival	−0.387	0.231	−0.223	−1.469	0.102
IGFBP-3	−3.590	2.006	−0.235	−1.790	0.081
Mullen at 6 months	0.298	0.114	0.333	2.607	0.018
OFC change at 6 months	7.000	3.319	0.280	2.109	0.031
	Non-verbal IQ (*R* ^2^ = 0.324, *F*(3,44) = 6.546, *p* = 0.001)				
	*B*	SE (B)	*β*	*t*	*p*
Age at arrival	−0.319	0.211	−0.204	−1.514	0.138
Mullen at 6 months	0.365	0.117	0.407	3.124	0.003
Height-for-age at arrival	2.814	1.797	0.212	1.566	0.125

Non-verbal IQ at 30 months was correlated with: age at arrival, height-for-age at arrival, and Mullen at 6 months (Table 3). Inclusion of these predictors in a regression model (Table 4) resulted in a significant model, *R*^2^ = 0.324, *F*(3,44) = 6.546, *p* = 0.001. Height-for-age at arrival and Mullen at 6 months were positive predictors of non-verbal IQ at 30 months.

## Discussion

This study explored the relationship between changes in physical growth and general cognitive outcomes in post-institutionalized children adopted from Eastern Europe over the course of the first 30 months post-adoption. As was predicted, significant improvements in height and weight measures and cognitive scores were observed, with normalization of measures in both domains seen at the assessment at 30 months post-adoption. These findings correspond with findings from a previous study using a larger number of participants from this data set [10] and are consistent with the findings from studies that considered populations of post-institutionalized children adopted into Western countries [11]. When compared to outcomes of post-institutionalized children who are placed domestically into foster care systems, the outcomes for internationally adopted children are, overall, more positive [17]. Two possible factors may play a role in the discrepancy between the gains in cognitive development observed in these two groups.

Firstly, results from previous studies suggest that children from institutional care in countries with a higher level of socioeconomic development show lesser delays in intellectual development, whereas in countries with particularly low socioeconomic development, there is sometimes no discrepancy observed between the intellectual development of family-reared and institutionalized children [12]. Romanian children in foster care may have been living with families with reduced access to resources, such as financial, medical, or educational support. Consequently, these children received care that was less optimal than the care the IA children in our study received. Furthermore, parents adopting international children may exhibit another selection factor in that they research and carefully review their prospective child’s medical records, asking for help and support in providing care to meet their child’s needs. Thus, regardless of placement with an adoptive family or foster care family, international PI children adopted into the USA may show increased gains in cognitive development as a product of more available resources and improved quality of care.

The significant positive changes were determined in height and weight measures over the first 30 months post-adoption and, consistent with Miller et al. [7], children who were smaller and younger at the time of their arrival showed more catch-up in height and weight at 30 months post-arrival. The association between the increase in weight and the increase in height is typically mediated by recovery of growth hormone secretion [7]. However, despite a significant change in mean values for OFC between the baseline and the 6-month assessments, there was not a significant change in the means for OFC after 6 months post-arrival. Furthermore, slower recovery of OFC was associated with poorer verbal IQ performance. Potentially, the window for recovery for physical growth in height and weight is more prolonged than that for recovery in OFC. This could explain why we often do not see as complete recovery in OFC measures as in other physical growth measures [11]. In order to promote optimal cognitive functioning, it will be important to continue investigating the factors involved in changes and recovery in OFC.

Consistent with previous findings, positive changes in the means between the baseline and the 6-month assessments of IGFBP-3 SD values and in IGF-1 levels were not significant [10]. Our findings did not indicate any association between these growth factors and a child’s height and weight status 30 months post-arrival. As it was previously suggested, other factors, such as nutrition and a highly enriched environment, may have a positive impact on both the GH system and catch-up growth [10].

Most of the children had IQ scores within normal range at the time of the 30-month follow-up assessment. In the analysis, we found several significant predictors of the general cognitive functioning at 30 months post-adoption including a child’s general developmental status at 6 months. It was found to be a predictor of both non-verbal and verbal scores at 30 months post-arrival. Age at arrival was correlated with cognitive outcomes at 30 months post-adoption but was not a significant predictor for either non-verbal IQ or verbal IQ when adjusted for other covariates. Child’s age at arrival was used as an approximation of the length of adverse experience prior to adoption in the analysis.

Initially, we hypothesized that a greater degree of adversity, defined as more initial stunting, would be associated with poorer general cognitive outcomes. Consistent with this hypothesis, a child’s initial height was found to be a significant predictor of non-verbal IQ. Children with more optimal height at arrival had higher non-verbal IQ measures. It was proposed previously that a child’s initial height status is a marker of allostatic load in this population and is a more sensitive predictor than age at adoption of the extent to which the child’s developmental trajectory has been compromised [5].

Contrary to our hypothesis, we did not find an association between initial status and changes in the growth factor IGF-1 and cognitive functioning at 30 months post-adoption. Changes in IGF-1 could have occurred rapidly between the time of arrival and the actual collection of blood in the clinic a few days or weeks later given that IGF-1 has the ability to change much faster in response to better circumstances than IGFBP-3 [7]. While we did not find an association with IGF-1, we did find that children with lower initial IGFBP-3 SD scores had higher verbal IQ scores at 30-months post-arrival. For verbal IQ performance, IGFBP-3 was found to be an independent significant predictor, along with initial developmental status and changes in OFC over the first 6 months. These findings are in line with Miller et al. [7], which illustrated a negative association with initial IGFBP-3 levels and physical growth status post-arrival.

One possible explanation for these findings is that low IGFBP-3 levels found in response to early adversity serve as a protective mechanism. In this scenario, low levels of IGFBP-3 potentially slow down or halt certain aspects of brain development, impacting both physical growth and general cognitive domains. Once in a more nurturing environment, where developmentally appropriate stimuli and nutrition are available, it resumes a more normal pattern of development. Since IGFBP-3 controls the local release of IGF-1, lower values of IGFBP-3 may allow higher levels of active or free IGF-1 to affect target tissues. This impact of IGFBP-3 in neuronal tissue may regulate neuronal growth and/or differentiation ultimately leading to improvement in cognitive function. Furthermore, IGFBP-3 may also be one of the mechanisms that underlie the more positive trajectory in white matter growth observed in PI children after placement in a family environment [23]. Thus, building upon these findings, our data suggest that IGFBP-3 may represent an important connection between linear growth, brain growth, and cognitive development that helps explain a connection between growth and cognitive development in post-institutionalized children. Future research will need to address this hypothesis and explore whether young children who are capable of effectively shutting down during times of early adversity show increased recovery in their neurodevelopment.

One of the limitations of this study is the lack of control of variability in the histories of the participants. Although most spent the majority of their early life in institutional care, we had limited knowledge regarding their prenatal development and genetic makeup. We were able to control for a prenatal factor, the exclusion of children at high risk of FASD, which could have impacted cognitive outcomes. We also excluded participants that were not medically healthy enough to complete testing. However, we cannot fully speak to the possible effects other aspects of the prenatal environment or individual differences could have had on the results. Future investigations could explore protective factors and individual differences as they relate to post-adoption outcomes. Another limitation is that the growth factor measures were not available at the 30-month post-arrival assessment. The small size of our sample potentially had an effect on multiple aspects of the findings. For example, a facet of this study explored the relationship between parental education and recovery in the general cognitive domain. Although a categorical analysis did not reveal a significant association between the level of parental education and child’s cognitive functioning, it is possible that an effect was precluded by the size of our sample. In the future, investigations with a larger sample size could continue to consider the impact of parental education and other aspects of post-adoption environment on positive changes post-adoption in multiple domains.

## Conclusions

Many of the internationally adopted children in this study arrived with severe developmental delays but showed substantial catch-up in physical growth and general cognitive status. Positive changes in the general cognitive functioning was found to be associated with the degree to which a child’s initial developmental status is compromised and the degree of early adversity, manifested both as compromised initial height and growth hormone system functioning.

This study provides us with guidelines for clinical practice with this population. Children who arrive stunted and who do not show a normal rate of catch-up growth by 6 months post-adoption should be seen by a medical specialist to provide appropriate early interventions [10]. Moreover, the physical growth and neurodevelopmental trajectories should be monitored in these children, and they should be referred for early intervention services as needed. Given that a child’s developmental status within the first 6 months post-arrival is a sensitive predictor of general cognitive functioning, a developmental evaluation is essential for early identification of children at risk and referral for early intervention

More than 681,000 children in the USA currently reside in temporary or foster care and their experiences and environments are similar to those of children in institutional care [29]. Consequently, the findings from this study are relevant to a greater population of at risk children. Comparing these similar yet distinct groups in future investigations can help to further understand the mechanisms and factors involved in promoting recovery post-adoption. Clinically, this leads to the development of more targeted interventions and the prospect of these children overcoming early adversity and reaching their full neurodevelopmental potentials.

## Abbreviations

BEIP: Bucharest Early Intervention Project
GH-IGF-1: growth hormone-insulin-like growth factor-I axis
IGFBP-3: insulin-like growth factor binding protein-3
PI: post-institutionalized

## References

[1] The Leiden Conference on the Development and Care of Children without Permanent Parents (2012). The development and care of institutionally reared children. Child Development Perspectives.

[2] UNAIDS, UNICEF, USAID (2004). Children on the brink 2004: a joint report of new orphan estimates and a framework for action.

[3] Bureau of Consular Affairs, U.S. Department of State. Intercountry Adoption. In: 2013. http://adoption.state.gov/about_us/statistics.php. Accessed 08/09 2013.

[4] Gunnar MR, van Dulmen MH, International Adoption Project Team (2007). Behavior problems in postinstitutionalized internationally adopted children. Dev Psychopathol.

[5] Johnson DE, Guthrie D, Smyke AT, Koga SF, Fox NA, Zeanah CH (2010). Growth and associations between auxology, caregiving environment, and cognition in socially deprived romanian children randomized to foster vs ongoing institutional care. Arch Pediatr Adolesc Med.

[6] Loman MM, Wiik KL, Frenn KA, Pollak SD, Gunnar MR (2009). Postinstitutionalized children’s development: growth, cognitive, and language outcomes. J Dev Behav Pediatr.

[7] Miller BS, Kroupina MG, Iverson SL, Masons P, Narad C, Himes JH (2009). Auxological evaluation and determinants of growth failure at the time of adoption in Eastern European adoptees. J Pediatr Endocrinol Metab.

[8] Johnson DE, Gunnar MR (2011). Growth failure in institutionalized children. Monogr Soc Res Child Dev.

[9] Baldini S, Restani L, Baroncelli L, Coltelli M, Franco R, Cenni MC (2013). Enriched early life experiences reduce adult anxiety-like behavior in rats: a role for insulin-like growth factor 1. J Neurosci.

[10] Miller BS, Kroupina MG, Mason P, Iverson SL, Narad C, Himes JH (2010). Determinants of catch-up growth in international adoptees from Eastern Europe. Int J Pediatr Endocrinol.

[11] van IJzendoorn MH, Bakermans-Kranenburg MJ, Juffer F (2007). Plasticity of growth in height, weight, and head circumference: meta-analytic evidence of massive catch-up after international adoption. J Dev Behav Pediatr.

[12] van IJzendoorn MH, Luijk MP, Juffer F (2008). IQ of children growing up in children’s homes: a meta-analysis on IQ delays in orphanages. Merrill-Palmer Quarterly (1982-).

[13] Nelson CA, Zeanah CH, Fox NA, Marshall PJ, Smyke AT, Guthrie D (2007). Cognitive recovery in socially deprived young children: the bucharest early intervention project. Science.

[14] Smyke AT, Koga SF, Johnson DE, Fox NA, Marshall PJ, Nelson CA (2007). The caregiving context in institution-reared and family-reared infants and toddlers in Romania. J Child Psychol Psychiatr.

[15] van IJzendoorn MH, Palacios J, Sonuga-Barke EJ, Gunnar MR, Vorria P, McCall RB (2011). Children in institutional care: delayed development and resilience. Monogr Soc Res Child Dev.

[16] Sheridan MA, Fox NA, Zeanah CH, McLaughlin KA, Nelson CA (2012). Variation in neural development as a result of exposure to institutionalization early in childhood. Proc Natl Acad Sci.

[17] Fox NA, Almas AN, Degnan KA, Nelson CA, Zeanah CH (2011). The effects of severe psychosocial deprivation and foster care intervention on cognitive development at 8 years of age: findings from the Bucharest Early Intervention Project. J Child Psychol Psychiatry.

[18] Beckett C, Castle J, Rutter M, Sonuga-Barke EJ (2010). Institutional deprivation, specific cognitive functions, and scholastic achievement: English and Romanian Adoptee (ERA) study findings. Monogr Soc Res Child Dev.

[19] Hearst MO, Himes JH, Johnson DE, Kroupina M, Syzdykova A, Aidjanov M (2014). Growth, nutritional, and developmental status of young children living in orphanages in Kazakhstan. Infant Mental Health J.

[20] Riikonen R (2006). Insulin-like growth factor delivery across the blood–brain barrier. Chemotherapy.

[21] Webb EA, O’Reilly MA, Clayden JD, Seunarine KK, Chong WK, Dale N (2012). Effect of growth hormone deficiency on brain structure, motor function and cognition. Brain.

[22] Gunnell D, Miller LL, Rogers I, Holly JM (2005). Association of insulin-like growth factor I and insulin-like growth factor-binding protein-3 with intelligence quotient among 8- to 9-year-old children in the Avon Longitudinal Study of Parents and Children. Pediatrics.

[23] Bick J, Zhu T, Stamoulis C, Fox NA, Zeanah C, Nelson CA (2015). Effect of early institutionalization and foster care on long-term white matter development: a randomized clinical trial. JAMA Pediatrics.

[24] Astley S (2003). FAS facial photographic analysis software.

[25] Mullen EM (1995). Manual for the Mullen Scales of Early Learning.

[26] Roid GH (2003). Stanford-Binet Intelligence Scales, Fifth Edition, Examiner’s Manual.

[27] Roche AF, Mukherjee D, Guo SM, Moore WM (1987). Head circumference reference data: birth to 18 years. Pediatrics.

[28] Center for Disease Control and Prevention. Atlanta, GA. 2015 http://www.cdc.gov/growthcharts/cdc_charts.htm. Accessed 08/19/2015.

[29] U.S. Department of Health and Human Services, Administration for Children and Families. Administration on Children, Youth and Families, Children’s Bureau. Child maltreatment. 2011. 2012

